# Hypoxia Inducible Factor (HIF)-1 Coordinates Induction of Toll-Like Receptors TLR2 and TLR6 during Hypoxia

**DOI:** 10.1371/journal.pone.0001364

**Published:** 2007-12-26

**Authors:** Johannes Kuhlicke, Julia S. Frick, Julio C. Morote-Garcia, Peter Rosenberger, Holger K. Eltzschig

**Affiliations:** 1 Department of Anesthesiology and Intensive Care Medicine, Tübingen University Hospital, Tübingen, Germany; 2 Department of Microbiology, Tübingen University Hospital, Tübingen, Germany; 3 Mucosal Inflammation Program, Department of Anesthesiology, and Perioperative Medicine, University of Colorado Health Science Center, Denver, Colorado, United States of America; Federal University of São Paulo, Brazil

## Abstract

**Background:**

During acute infection and inflammation, dramatic shifts in tissue metabolism are typical, thereby resulting in profound tissue hypoxia. Therefore, we pursued the hypothesis, that tissue hypoxia may influence innate immune responses by transcriptional modulation of Toll-like receptor (TLRs) expression and function.

**Methodology/Principal Findings:**

We gained first insight from transcriptional profiling of murine dendritic cells exposed to hypoxia (2% oxygen for 24 h). While transcript levels of other TLRs remained unchanged, we found a robust induction of TLR2 (2.36±0.7-fold; P<0.05) and TLR6 (3.46±1.56-fold; P<0.05). Additional studies in different cells types and cell-lines including human dendritic cells, monocytic cells (MM6), endothelia (HMEC-1) or intestinal epithelia (Caco-2) confirmed TLR2 and TLR6 induction of transcript, protein and function during hypoxia. Furthermore, analysis of the putative TLR2 and TLR6 promoters revealed previously unrecognized binding sites for HIF-1, which were shown by chromatin immunoprecipitation to bind the pivotal hypoxia-regulating transcription factor HIF-1alpha. Studies using loss and gain of function of HIF-1 confirmed a critical role of HIF-1alpha in coordinating TLR2 and TLR6 induction. Moreover, studies of murine hypoxia (8% oxygen over 6 h) showed TLR2 and TLR 6 induction in mucosal organs in vivo. In contrast, hypoxia induction of TLR2 and TLR6 was abolished in conditional HIF-1α mutant mice.

**Conclusions/Significance:**

Taking together, these studies reveal coordinated induction of TLR2 and TLR6 during hypoxia and suggest tissue hypoxia in transcriptional adaptation of innate immune responses during acute infection or inflammation.

## Introduction

Microorganisms that invade a vertebrate host are initially recognized by the innate immune system through germline-encoded pattern-recognition receptors [1]. Innate defense mechanisms rely heavily on such signaling pathways to alert the immune system of the presence of the invading pathogens. As a group of pattern-recognition molecules, Toll-like receptors (TLRs) have evolved into a central role during such responses. As such, mammalian TLRs represent a family of at least 12 membrane proteins that trigger innate immune responses through nuclear factor-κB (NF-κB)-dependent and interferon (IFN)-regulatory factor-dependent signalling pathways [2]. TLRs are evolutionarily conserved molecules and were originally identified in vertebrates on the basis of their homology with “Toll”, a molecule that stimulates the production of antimicrobial proteins in *Drosophila melanogaster* [3]. Mammalian TLRs have been functionally characterized and distinguished mainly on the basis of their stimulation by different ligands [4]. As such, the TLR-family members are pattern-recognition receptors (PRRs) that recognize lipid, carbohydrate, peptide and nucleic-acid structures that are broadly expressed by different groups of microorganisms [2]. In addition to their role in innate immune responses during infection, the recognition of endogenous ligands by TLRs is now thought to have an important role also in the regulation of inflammation, both in infectious and non-infectious diseases [2].

In this context, acute sites of inflammation or infection are characterized by dramatic shifts in tissue metabolism [5]. These changes include increased consumption of oxygen by residential or recruited inflammatory cells, and diminished availability of oxygen due to thrombosis or inflammation of the vascular support system, resulting in profound hypoxia [6]–[8]. Such shifts in tissue metabolism result, at least in part, from massive recruitment of inflammatory cell types or pathogens [5], [9], [10]. As such, studies of innate immune responses have found a central role of hypoxia–elicited signaling pathways in inflammatory immune responses. For example, a very elegant study of inflammatory response in mice with conditional knockouts of the hypoxia responsive transcription factor hypoxia inducible factor (HIF)-1α revealed profound impairment of myeloid cell aggregation, motility, invasiveness, and bacterial killing [11]. Taken together, these studies demonstrate a role for HIF-1α in the direct regulation of survival and function in the inflammatory microenvironment.

At the tissue and cellular level, hypoxia induces an array of genes pivotal to survival in low oxygen states. As a global regulator of oxygen homeostasis, the αβ heterodimeric transcription factor HIF-1 facilitates both oxygen delivery and adaptation to oxygen deprivation [12]. HIF-1 is a member of the Per-ARNT-Sim (PAS) family of basic helix-loop-helix (bHLH) transcription factors. HIF-1 activation is dependent upon stabilization of an O_2_-dependent degradation domain of the α subunit and subsequent nuclear translocation to form a functional complex with HIF-1β and cofactors such as CBP and its ortholog p300 [13]. Under conditions of adequate oxygen supply, iron and oxygen dependent hydroxylation of two prolines (Pro564 and Pro 402) within the oxygen-dependent degradation domain (ODD) of HIF-1α initiates the association with the von Hippel-Lindau tumor suppressor protein (pVHL) and rapid degradation via ubiquitin-E3 ligase proteasomal targeting [12]. A second hypoxic switch operates in the carboxy terminal transactivation domain of HIF-1α ~ Here, hypoxia blocks the hydroxylation of asparagine-803 so facilitating the recruitment of CBP/p300 [14]. When levels of oxygen fall below a critical threshold (hypoxia), the lack of PHD substrate (oxygen) results in the accumulation of HIF̃α, which then associates with HIF−β. The HIF heterodimer translocates to the nucleus where it is made available to activate HIF-bearing gene promoters. Genes induced by HIF-1 include those necessary for cell, tissue and whole animal adaptive responses to hypoxia [12]. These proteins include enzymes involved in anaerobic metabolism, the angiogenic cytokine vascular endothelial growth factor (VEGF) and inducible nitric oxide synthase [12].

In the present study, we pursued the hypothesis that tissue hypoxia–as occurs during inflammation or infection–may modulate PRR-dependent signaling pathways of the innate immune system. For this purpose, we screened mammalian TLR expression during normoxic or hypoxic conditions. Serendipitously, these studies revealed a central role of HIF-1 in the coordinated induction of TLR2 and TLR6, which are known for their functional interaction in the cellular response to different microorganisms [15], [16].

## Results

### Modulation of TLR signaling by hypoxia

**Figure 1:**
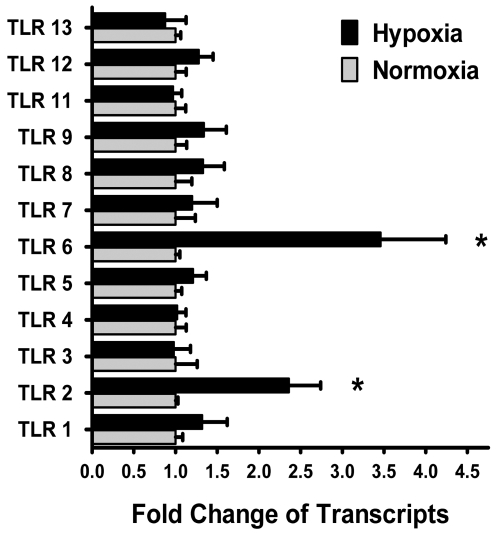
Influence of hypoxia on TLR mRNA expression in murine dendritic cells. Isolated murine bone-marrow-derived dendritic cells (BMDCs) were exposed to normoxia or hypoxia for 24 hours. Total RNA was isolated, and quantitative mRNA levels of TLR1-9 and TLR11-13 were assessed by real-time RT-PCR. Data were calculated relative to ß-actin and expressed as fold change relative to normoxia±SEM and transcript levels in normoxic BMDCs were normalized to 1. Results are derived from three different experiments (*p<0.05, significant differences from normoxia).

Sites of infection and inflammation are characterized by dramatic shifts in metabolic supply and demand, thereby leading to profound tissue hypoxia [5]. Given the association of hypoxia with tissue-infections and the predominant role of TLR-signaling in regulating host-cell responses during infections with human pathogens [2], we pursued transcriptional responses of TLRs during hypoxia. As first step in the line of these experiments, we examined the influence of hypoxia on expressional levels of mammalian TLRs (TLR1-9 and 11-13) in vitro, using a cellular model of murine dendritic cells. For this purpose, we isolated bone-marrow-derived dendritic cells (BMDCs) from C57BL/6×129SV mice and exposed them ex vivo to normoxia or normobaric hypoxia (2% oxygen, 24h). Using real-time RT-PCR analysis, we found selective induction of mRNA levels of TLR2 (2.360±0.3810, P<0.05) and TLR6 (3.460±0.7820, p<0.05) with hypoxia exposure in comparison to normoxia (Fig. 1). In contrast, transcript levels of other TLRs remained unchanged with hypoxia exposure. Due to the fact that TLR10 expression has not been detected in murine BMDCs [17] we studies modulation of TLR 10 transcript in human microvascular endothelia (HMEC-1) and human intestinal epithelia (CaCo-2). These studies revealed no changes of TLR10 transcript with hypoxia (data not shown). Taken together, these studies show a robust and selective induction of TLR2 and TLR6 coordinated by hypoxia.

### Hypoxia induces TLR2 mRNA and protein and enhances TLR2 signaling effects

**Figure 2:**
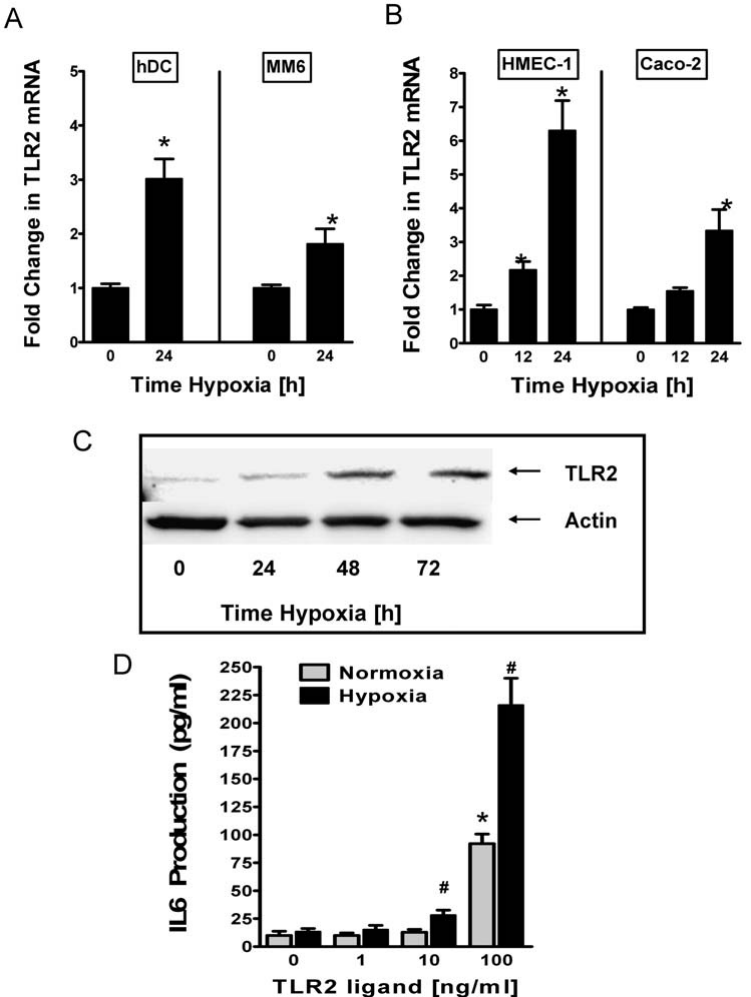
TLR2 transcript, protein and function during hypoxia.

Based on the above observation of coordinated induction of TLR2 and TLR6 with hypoxia, we next pursued additional details of TLR2 induction with hypoxia using different cellular models. Therefore, we exposed human dendritic cells (hDCs), monocytic cells (MM6), endothelia (HMEC-1) and epithelia (Caco-2) over indicated time periods to hypoxia (2% oxygen), isolated RNA and performed real-time RT-PCR to determine TLR2 transcript levels. Consistent with our studies of murine dendritic cells, we found a robust induction of TLR2 transcript in all examined cells (Figure 2 A, B). For example, real-time RT-PCR analysis revealed prominent induction of TLR2 mRNA expression in hDCs (3.012±0.3002, p<0.01) and MM6 cells (1.812±0.4875, p<0.05) after 24h exposure to hypoxia. Similarly, TLR2 transcript was significantly elevated after 12 h of hypoxia exposure of HMEC-1 (2.170±0.2944, p<0.05) or Caco-2 cells (1.543±0.1009, p<0.05). We next pursued induction of TLR2 protein levels by Western blot analysis. For this purpose, HMEC-1 were grown to full confluency and exposed to hypoxia (0, 24, 48 and 72 h; pO_2_ 20 torr). Indeed, we found increases of TLR2 protein in HMEC-1 after different time points of hypoxia exposure (Fig. 2C). As next step, we studied functional consequences of TLR2 induction by hypoxia. Previous studies had shown specific activation of TLR2 by N-Palmitoyl-bis(palmitoyloxy)-propyl-cysteinyl-seryl-Lys4 (P3C) leading to TLR2-signaling dependent activation of NF-κB and the production of pro-inflammatory cytokine IL6 [18]. For that purpose we stimulated normoxic or post-hypoxic HMEC-1 cells (48 h at 2% oxygen) with the TLR2 agonist P3C and measured IL6 secretion into the supernatant. These studies revealed that TLR2-dependent release of IL6 was dramatically enhanced in post-hypoxic HMEC-1 (Fig. 2D, p<0.01 with 100 ng/ml P3C). Taken together these studies confirm that TLR2 transcript, protein and function are induced by hypoxia.

### Hypoxia induction of TLR6 transcript, protein and function

**Figure 3:**
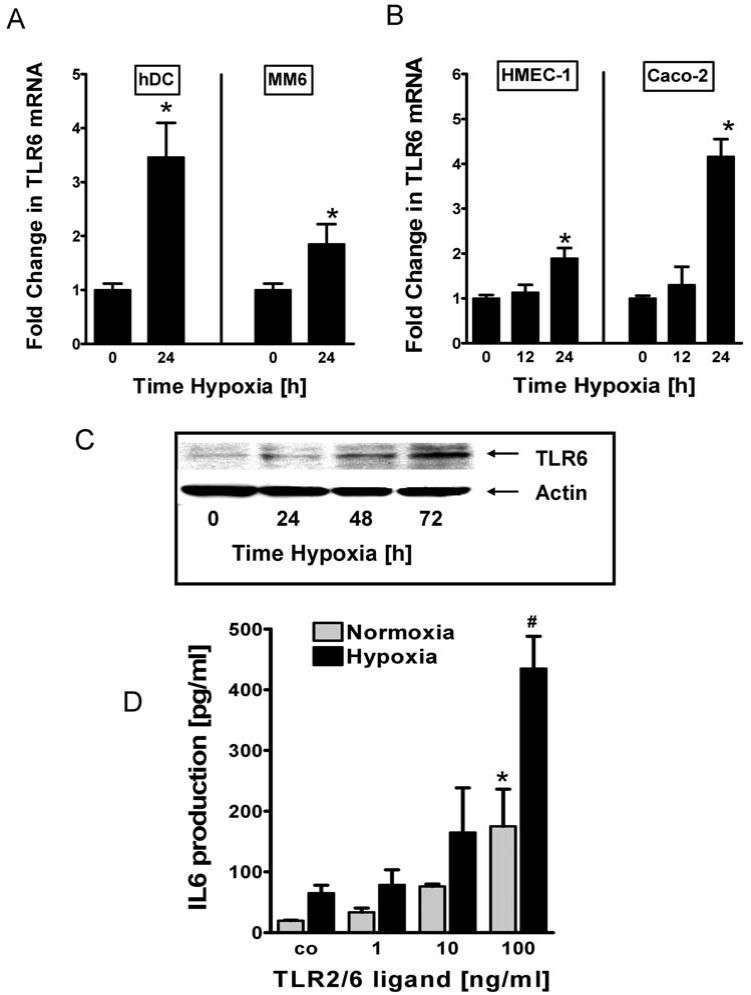
TLR6 transcript, protein and function during hypoxia. *A* and *B*, Quantification of TLR6 transcripit levels in freshly purified blood DCs, MM6 cells, confluent HMEC-1 monolayers and confluent Caco-2 monolayers. Cells were exposed to normoxia and hypoxia for indicated time points. Total RNA was isolated and TLR6 mRNA levels were determined by real-time RT-PCR. Data were calculated relative to ß-actin and expressed as fold change relative to normoxia±SEM, where transcript levels in normoxic cells were normalized to 1. Results are derived from three different experiments. *, significant differences from normoxic cells (p<0.01). *C*, Confluent HMEC-1 cells were grown to confluence and exposed to indicated periods of hypoxia. Result depicts a representative TLR6 Western blot from three separate experiments. The same blot was probed for ß-actin expression as a control for protein loading. *D*, Same amount of HMEC-1 cells were grown to confluence in 24-well plates. Cells were then stimulated with TLR2/6 agonist (FSL-1) at the indicated concentrations and exposed to hypoxia or normoxia for 24 h. After 24 h, generation of IL6 was measured by ELISA in the cell supernatant. Data are mean±SEM from 3 separate replicates. *, significant differences from untreated cells (p<0.05); #, significant differences from normoxia and untreated cells (p<0.05).

After having shown induction of TLR2 with hypoxia, we next studied additional details of TLR6 induction during hypoxia. In fact, TLR6 can function as co-receptor and heterodimerizes with TLR2 to form preexisting cell surface receptors on different cell types [19]. Such TLR2/TLR6 complexes play an important role in innate immune responses by recognizing microbial compounds including lipoteichoic acids from gram-positive bacteria [20] or fungal zymosan [21]. In order to confirm TLR6 inducibility by hypoxia, we exposed human DCs, MM6, HMEC-1 or Caco-2 epithelia to hypoxia (2% oxygen) and measured changes in TLR6 transcript level by real-time RT-PCR. Similar to the above findings of TLR2 induction by hypoxia, real-time RT-PCR confirmed hypoxia induction of TLR6 in all cell lines studied (Figure 3A and B). For example, TLR6 transcript levels were dramatically elevated following 24 h of hypoxia exposure in Caco-2 cells (4.080±0.4446, p<0.01). We next verified these findings on a protein level by measuring TLR6 expression by Western blot analysis. Here, we exposed confluent HMEC-1 monolayers to hypoxia over 0 to 72h to hypoxia. Consistent with our studies of TLR6 transcript, we found a robust induction of TLR6 protein with hypoxia (Figure 3C). As next step we studied TLR signaling effects during normoxia or hypoxia. Here, we used a specific ligand for the TLR2/TLR6 complex (FSL-1, range 1–100 ng/ml) and measured IL6 concentration in the HMEC-1 pre-exposed to normoxia or 24 h of hypoxia (2% oxygen). In fact, FSL-1 stimulated IL6 production was significantly enhanced in post-hypoxic HMEC-1 (Fig. 3D), showing that hypoxia enhances signaling through the TLR2/6 complexes. These findings confirms induction of TLR6 transcript, protein and function by ambient hypoxia.

### Role of hypoxia inducible factor (HIF)-1 in TLR2 regulation

**Figure 4:**
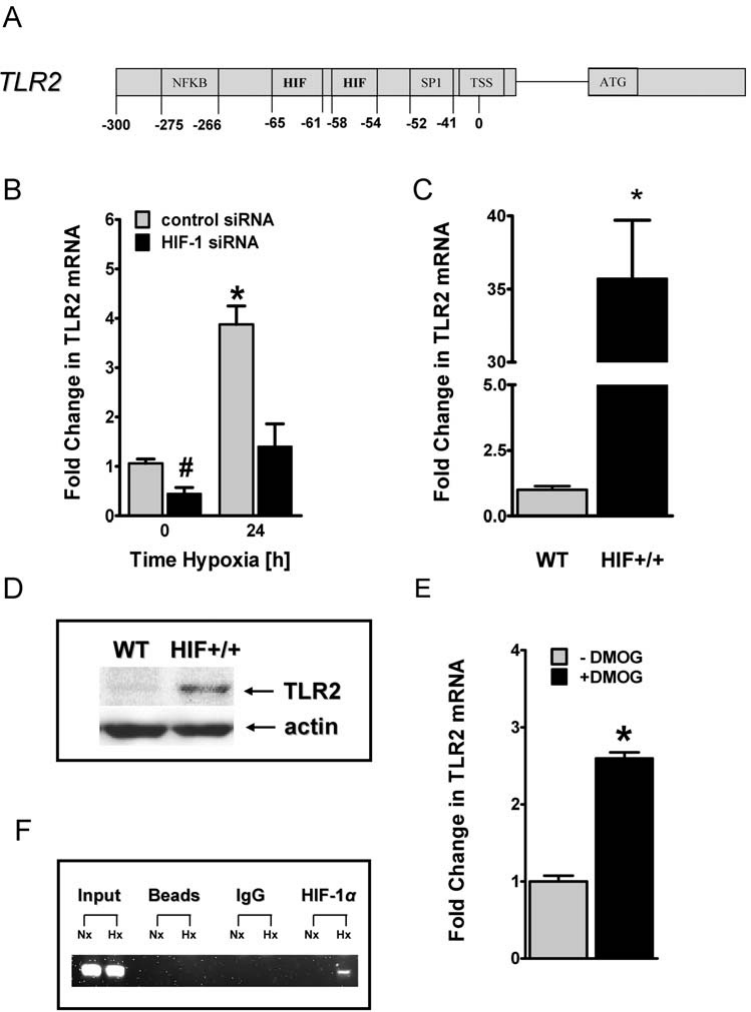
Influence of hypoxia inducible factor (HIF)-1α on TLR2 expression during hypoxia. *A*, Map of TLR2 promoter region showing positions of the putative HIF binding sites and the binding site for NFκB and SP1 relative to the transcription start site (TSS). *B*, Stable transfected HMEC-1 monolayers containing either HIF-1α siRNA or control-siRNA were exposed to normoxia or hypoxia for indicated time points. Total RNA was isolated, and 1 µg of RNA was transcribed into first strand cDNA. Relative expressional levels of TLR2 transcript were compared to normoxic controls by real-time RT-PCR. Data were calculated relative to internal control gene (ß-actin), and are expressed as fold change over normoxia±SEM, *, significant differences from normoxia and control cells (p<0.05). Results are derived from three different experiments in each condition. *C*, Total RNA of normoxic monolayers of either wildtype (WT) or oxygen-stable HIF-1α expressing (HIF^+/+^) HMEC-1 cells was isolated and real-time RT-PCR was performed as described above. *, significant differences from wildtype cells (p<0.001). *D*, Western blot analysis of TLR2 protein of normoxic HMEC-1 wild type (WT) and oxygen-stable HIF-1α expressing (HIF+/+) cells. The same blot was probed for ß-actin expression as a control for protein loading. *E*, HMEC-1 monolayers were treated with 1mM of dimethyloxalylglycine (DMOG), following measurement of TLR2 transcript levels by real-time RT-PCR as described above. *, significant differences from untreated cells (p<0.001). *F*, ChIP assay was utilized to examine HIF-1α binding to the TLR2 promoter in normoxic and hypoxic HMEC-1 cells. Reaction controls included samples precipitated with protein G sepharose beads alone (beads), immunoprecipitations using a nonspecific igG monoclonal antibody (IgG) and PCR performed using HMEC-1 DNA (input). An example of three experiments is shown.

In an attempt to gain specific insight into the mechanisms of TLR2 induction, we began examining induction pathways from hypoxia response genes. In the course of our experiments, we identified two previously unappreciated HIF-1 binding sites in the TLR2 gene promoter (DNA consensus motif 5′-CCGTG-3′ located at positions –54 to –58 and -61 to -65 relative to the major transcription start site, Fig. 4A) [22]. To study a functional contribution of HIF-1α in hypoxia induction of TLR2, we next pursued HIF-1 loss and gain of function. As shown in Figure 4B, baseline transcript levels are significantly repressed and hypoxia inducibility of TLR2 transcript is attenuated when using a previously characterized HMEC-1 line with stable siRNA repression of HIF-1α compared to control transfected cells [23]. In addition, we studied TLR2 expression in a previously characterized HMEC-1 line with oxygen stable overexpression of HIF-1 [23]. As shown in Figure 4, normoxic expression of HIF-1α is associated with dramatic increases of TLR2 transcript (35.7±69-fold increase, P<0.001). These findings could be confirmed on a protein level by Western blot analysis (Fig. 4D). Next, we used the HIF-1 activator dimethyloxalylglycine (DMOG, a non specific inhibitor of prolylhydroxylases) [24], [25]. Similar to HIF-overexpression, pretreatment of HMEC-1 with DMOG was associated with induction of TLR2 transcript levels (Figure 4E). As last step, we determined whether this region of the TLR2 promoter binds HIF-1α. For these purposes, we used chromatin immunoprecipitation (ChIP) to study HIF-1α binding in live cells. As shown in Figure 4F, ChIP analysis of nuclei derived from HMEC-1 cells revealed a prominent band in hypoxic but not normoxic samples. No bands were evident in beads only or control IgG immunoprecipitates, and input samples (preimmunoprecipitation) revealed the predictable band under conditions of both hypoxia and normoxia. Such results indicate that hypoxia induces HIF-1α binding to the TLR2 promoter region. Together, these results suggest that TLR2 induction by hypoxia is mechanically determined, at least in part, by HIF-1.

### HIF-1α in TLR6 induction during hypoxia

**Figure 5:**
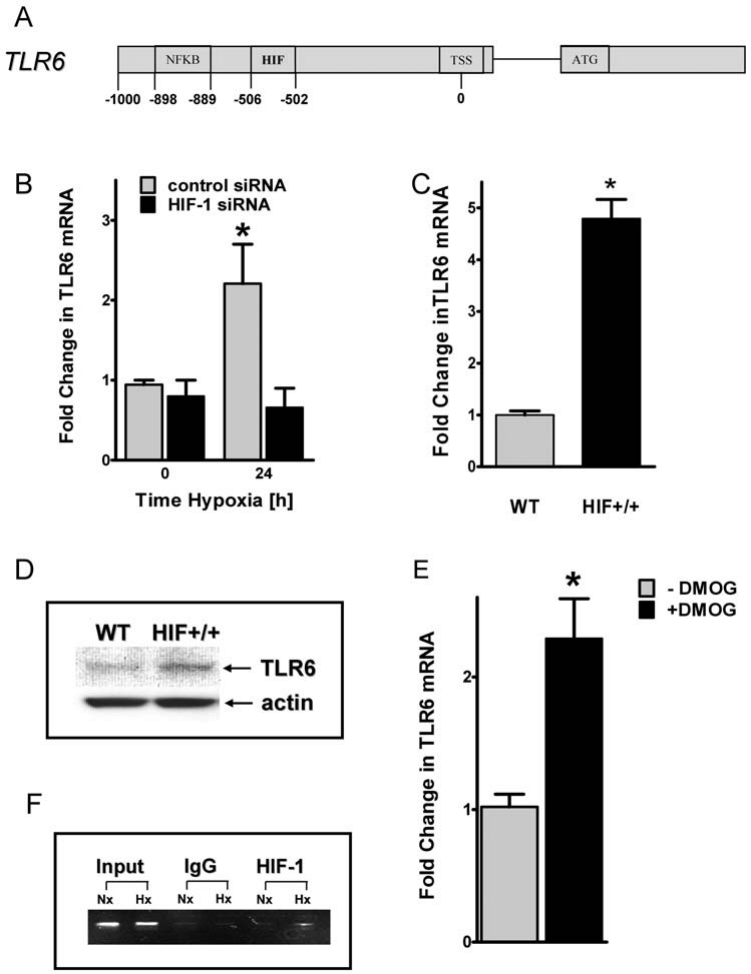
Influence of hypoxia inducible factor (HIF)-1α on TLR6 expression during hypoxia. *A*, Map of TLR6 promoter region showing positions of the putative HIF binding sites and the binding site for NFκB relative to the transcription start site (TSS). *B*, Stable transfected HMEC-1 monolayers containing either HIF-1α siRNA or control-siRNA were exposed to normoxia or hypoxia for indicated time points. Total RNA was isolated, and 1 µg of RNA was transcribed into first strand cDNA. Relative expressional levels of TLR6 transcripts were compared to normoxic controls by real-time RT-PCR. Data were calculated relative to internal control gene (ß-actin), and are expressed as fold change over normoxia±SEM, *, significant differences from normoxia and control cells. Results are derived from three different experiments in each condition. *C*, Total RNA of normoxic monolayers of either wildtype (WT) or oxygen-stable HIF-1α expressing (HIF^+/+^) HMEC-1 cells was isolated and realt-time RT-PCR was performed as described above. *, significant differences from wildtype cells. *D*, Western blot analysis of TLR6 protein of normoxic HMEC-1 wildtype (WT) and oxygen-stable HIF-1α expressing (HIF+/+) cells. The same blot was probed for ß-actin expression as a control for protein loading. *E*, HMEC-1 monolayers were treated with 1mM of dimethyloxalylglycine (DMOG) for 24 hours. Afterwards transcript levels of TLR6 where quantified by real-time RT-PCR as described above. *, significant differences from untreated cells. *F*, ChIP assay was utilized to examine HIF-1α binding to the TLR6 promoter in normoxic and hypoxic HMEC-1 cells. Reaction controls included immunoprecipitations using a nonspecific igG monoclonal antibody (IgG) and PCR performed using HMEC-1 DNA (input). An example of three experiments is shown.

Similar to our studies of mechanisms of TLR2 induction by hypoxia, we next attempted translational mechanisms of TLR6 induction during hypoxia. Similar to TLR2, we identified a previously unrecognized binding site for HIF-1 in the putative TLR6 gene promoter (DNA consensus motif 5′-CCGTG-3′ located at positions –502 to –506 relative to the transcription start site, Fig. 5A). Consistent with our studies of TLR2, HIF-1 loss and gain of function studies suggest a functional role of HIF-1α in TLR 6 induction. As shown in Figure 5B, hypoxia inducibility of TLR6 transcript is attenuated in HMEC-1 with stable siRNA repression of HIF-1α, while normoxic overexpression of HIF-1α (Figure 5C and D) or DMOG treatment (Figure 5E) is associated with increased TLR6 levels. Finally, ChIP analysis of the putative TLR6 promoter confirmed HIF-1α binding to the promoter region during hypoxia (Figure 5F). Taken together these results suggest HIF-1 in coordination of TLR2 and TLR6 induction by hypoxia.

### TLR2 and TLR6 are induced during ambient hypoxia *in vivo*

**Figure 6:**
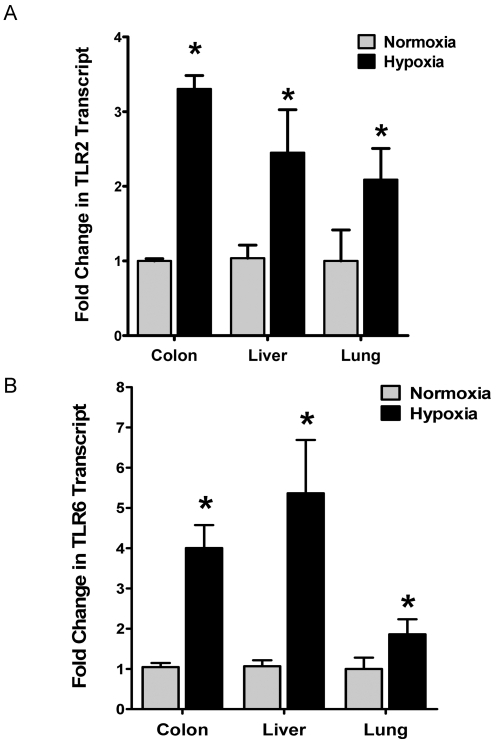
Expression of TLR2 and TLR6 during ambient hypoxia in vivo. *A* and *B*, Expression of TLR2 and TLR6 mRNA in normoxic or hypoxic organs. Tissue of Colon, Liver and Lung where harvested from mice after exposure to normoxia or normobaric hypoxia (8% O2, 92% N2 for 6h). Total RNA was isolated, and quantitative mRNA levels of TLR2 and TLR6 were assessed by real-time RT-PCR. Data were calculated relative to ß-actin and expressed as fold change relative to normoxia±SEM and transcript levels in normoxic organs were normalized to 1. Results are derived from six animals in each condition (*p<0.01, significant differences from normoxia).

**Figure 7:**
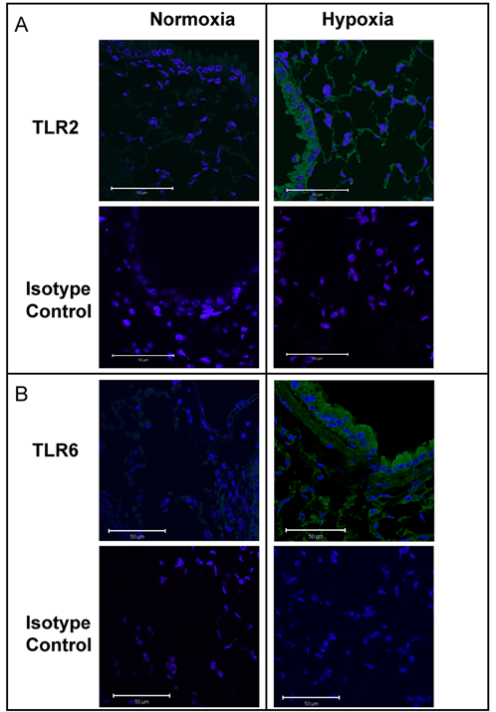
Pulmonary immunohistochemistry for TLR2 and TLR6 during hypoxia. *A* and *B*, Lungs from mice exposed to normoxia or normobaric hypoxia (8% O2, 92% N2 for 4h) were harvested, formalin-fixed and paraffin-embedded. Sections were stained with antibodies for TLR2 and TLR6 or isotype controls. This figure is representative of three experiments in each condition.

To confirm hypoxia induction of TLR2 and TLR6 in vivo, we utilized a previously described murine model of ambient hypoxia [26]–[30]. For this purpose, we exposed C57BL/6 mice over 6h to ambient hypoxia (8% oxygen), and examined TLR2 and TLR6 transcript levels in mucosal organs including the colon, liver and lungs. As shown in Figure 6A (TLR2) and 6B (TLR6), real-time PCR analysis revealed a significant increase of TLR2 and TLR6 transcript in all three examined organs. In addition, immunohistochemical staining of sections of lung tissues confirmed TLR2 (Figure 7A) and TLR6 (Figure 7B) induction in vivo. Taken together, these findings indicate that induction of TLR2 and TLR6 also occurs during ambient hypoxia *in vivo*.

#### Role of HIF-1α in TLR2 and TLR6 expression *in vivo*

**Figure 8:**
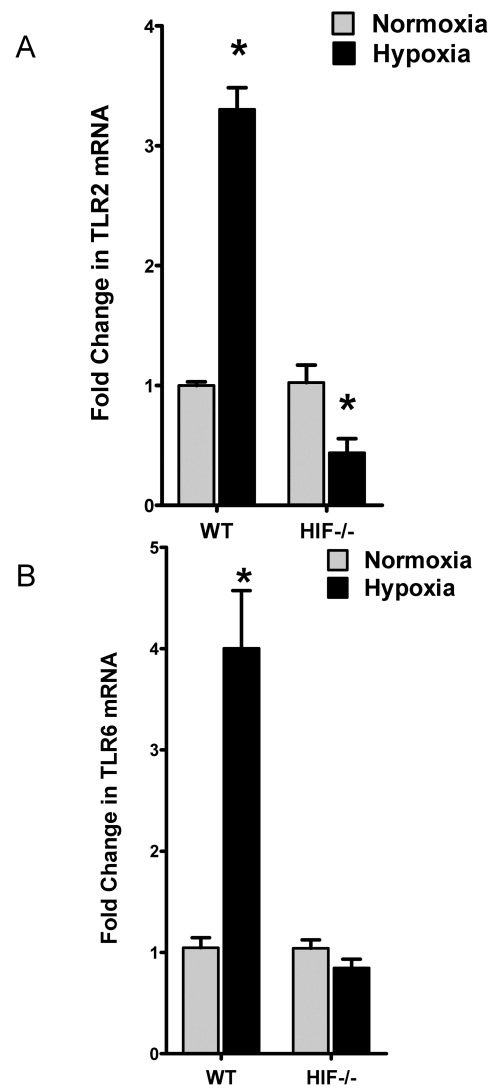
Role of hypoxia inducible factor (HIF)-1 in TLR2 and TLR6 expression during hypoxia in vivo. Real-time RT-PCR analysis of murine epithelial TLR2 and TLR6 mRNA in conditional HIF-1α mutant (HIF-/-) and littermate control (WT) animals subjected to normoxia or hypoxia. Data were calculated relative to ß-actin and are expressed as fold change over normoxia±SEM, where transcript levels of control animals were normalized to 1. *, significant differences from normoxic control animals (p<0.05). Results are derived from 8 animals in each condition.

As last step in the line of these experiments, we extended these findings of HIF-1–mediated induction of TLR2 and TLR6 expression into a genetic in vivo model. Here, we examined the influence of hypoxia on TLR2 and TLR6 transcript levels in intestinal epithelia derived from conditionally gene-targeted HIF-1α mice, in which intestinal epithelia lack detectable HIF-1α expression in >70% of cells [31]. As show in Figure 8A and 8B, TLR2 and TLR6 levels in mice expressing wild-type HIF-1α showed a normal pattern of hypoxia-associated induction of TLR2 and TLR6. Consistent with our hypothesis that HIF-1 transcriptionally induces TLR2 and TLR6, real-time PCR analysis revealed abolished induction of TLR2 and TLR6 transcript levels in intestinal epithelial derived from Hif1α mutant animals. Taken together, such findings support our *in vitro* findings and indicate the likelihood that HIF-1 directly regulates murine TLR2 and TLR6 expression.

## Discussion

Sites of inflammation and infection are characterized by significant changes in metabolic supply and demand. Such shifts frequently result in inflammation-associated tissue hypoxia. Due to the central role of the TLR-signaling system in the regulation of innate immune responses, both in infectious and non-infectious diseases [2], we pursued transcriptional effects of hypoxia on TLR expression patterns. A screen of mammalian TLRs for hypoxia responsiveness revealed a selective and robust induction of TLR2 and TLR6. Additional studies in different cellular models (dendritic cells, monocytes, endothelia or epithelia) consistently confirmed hypoxia-induction of TLR2 and TLR6. Using ChIP assays, cellular models of HIF-loss and gain of function as well as murine genetic models we could mechanistically determine HIF-1α in the coordination of this response. Taken together, these studies reveal modulation of TLR-dependent immune responses by hypoxia and confirm previous studies on a central role of HIF in the regulation of innate immunity [5], [11], [31]–[34].

Serendipitously, our studies revealed a coordinated induction of TLR2 and TLR6 by hypoxia, as both receptors owe each other a close functional relationship. In fact, a very elegant study on the coordination of TLR responses revealed that TLR2 and TLR6, together coordinate macrophage activation by Gram-positive bacteria and the yeast cell-wall particle, zymosan [21]. In fact, TLR6 and TLR2 are both recruited to the macrophage phagosome, where they recognize peptidoglycan, a Gram-positive pathogen component. By contrast, TLR2 recognizes another component, bacterial lipopeptide, without TLR6. The requirement for TLR cooperation is supported by the finding that TLR2 needs a partner to activate tumor necrosis factor-alpha production in macrophages. As such, these studies revealed that TLR2 forms functional pairs with TLR6 or TLR1, and this interaction leads to cytokine induction. The data suggest that TLRs sample the contents of the phagosome independent of the nature of the contents, and can establish a combinatorial repertoire to discriminate among the large number of pathogen-associated molecular patterns found in nature. As such, TLR2 seems to be the most promiscuous TLR receptor able to recognize the most diverse set of pathogen-associated patterns [19]. In fact, its promiscuity has been attributed to its unique ability to heterodimerize with other TLRs, particularly TLR1 and TLR6. Thus, it seems that TLR2 forms receptor clusters in response to different microbial ligands. In this context, a different study investigated TLR2 cell surface heterotypic interactions in response to different ligands as well as internalization and intracellular trafficking [19]. This study showed that TLR2/TLR6 heterodimers pre-exist and are not induced by the ligand. Upon stimulation by the specific ligand, these heterodimers are recruited within lipid rafts [19]. Thus, it is not surprising that transcriptional regulation of TLR2 and TLR6 are tightly linked together, while hypoxia-dependent induction of TLR2 and TLR6 is a coordinated response under the direct control of HIF-1α.

Only recently, many parallels between hypoxia and acute infection and inflammation have become obvious. For example, HIF-1 is essential for myeloid cell-mediated inflammation, bactericidal capacity of phagocytes [35] and mice with conditional knockouts of HIF-1 show profound impairment of myeloid cell aggregation, motility, invasiveness, and bacterial killing [11]. Similarly, HIF-1 has been identified as key activator of the inflammatory transcription factor NF-κB [36]. Similarly, recent studies have also found a critical role of HIF-1 during bacterial infections. For example, exposition of host cells to bacteria (*Bartonella henselae*) results in HIF-1 activation and vascular endothelial growth factor (VEGF) secretion *in vivo* and *in vitro* [37]. Similar findings were reported when macrophages were infected with group B streptococci [11], [35]. These studies highlight the role of HIF-1 in transcriptional regulation of infections with human bacterial pathogens. Moreover, HIF plays a critical role in inflammatory diseases, such as inflammatory bowel disease. Colonic epithelial cells are anatomically positioned to provide a selective barrier to luminal antigens and pathogens. Supported by a complex vasculature, this physiologically crucial barrier is a primary target for diminished blood flow and resultant tissue hypoxia. A number of studies have indirectly implicated hypoxia in mucosal inflammatory diseases such as colitis [38]–[41], and recent studies in murine models identified the epithelium as the central target of hypoxia during active inflammation [31]. As such, the existence of mucosal hypoxia during inflammation could be confirmed using 2-nitroimidazole compounds, which are retained in the absence of adequate oxygen levels and can be visualized using specific antibodies [42]. To further detail the physiologic implications of epithelial HIF-1, a very elegant study by Karhausen *et al.* utilized two mouse lines with intestinal epithelial-targeted expression of either mutant HIF-1α (constitutive repression of HIF-1) or mutant von Hippel-Lindau gene [31]. Studies of colitis in these mice revealed that epithelial “HIF-1 loss of function” correlated with more severe clinical symptoms (mortality, weight loss, colon length, intestinal epithelial permeability), whereas an increase in epithelial HIF-1 was protective for these individual parameters.

At present, it remains unclear, whether HIF-1 represents a protective host defense during inflammatory hypoxia, or is part of a pathogenic response elicited by invading pathogens or excessive inflammation. While recent studies in septic patients have demonstrated an anti-protective effect of the HIF-1 regulated-gene VEGF and suggest treatment of sepsis with VEGF-receptor antibodies [43], other studies of inflammation and infection have found a host-protective role of HIF-1. Such studies include a protective role of HIF in murine colitis [31], adenosine-dependent anti-inflammatory signaling pathways during hypoxia [26], [28], [30], [44]–[47], myeloid cell function [11] or bacterial capacity of phagocytes [35]. With regard to the present findings of coordinated induction of the TLR2/6 complex during hypoxia, it remains unclear if such changes in TLR signaling patterns represent a protective host-defense mechanism or can further exacerbate infection and inflammation associated tissue damage. However, recent studies suggesting a protective role of TLR2 signaling in experimental colitis [48]. In this study, oral treatment of murine colitis with a TLR2 ligand significantly suppressed mucosal inflammation and apoptosis by restoring tight junctional integrity of the intestinal epithelium in vivo. In this context, it is tempting to speculate that HIF-dependent induction of TLR2 and TLR6 may be part of hypoxia-elicited innate protection during epithelial inflammation and treatment with HIF-activators (such as DMOG) may be protective during colitis via TLR2 induction and signaling.

Taken together, the present studies reveal transcriptional-dependent induction of TLR2 and TLR6 during hypoxia. Based on ChIP analysis, cellular models of HIF-loss and gain of function and additional genetic studies using conditional deletion of HIF-1α, it appears likely that this response is coordinated by HIF-1α. As such, these studies highlight the central role of HIF-1 in the transcriptional control of signaling pathways in innate immune responses.

## Materials and Methods

### Cell culture

Human microvascular endothelial cells (HMEC-1) were cultured as described previously [23], [26], [28]–[30], [49]. Human colonic epithelial cells (Caco2) were grown and maintained as confluent monolayers in 75 cm^2^ growing flasks as previously described [50]. MM6 cells were cultured as described previously [51]. Prior to all experimental procedures, cultured cells were tested to rule out contamination with Mycoplasma spp.. Human dendritic cells (hDCs) were islotated from buffy coats of healthy blood donors as described previously [52]. BMDCs were isolated and cultured as described previously [53].

### Transcriptional Studies

Transcript levels were quantified by real-time reverse transcription-polymerase chain reaction (RT-PCR, iCycler; Bio-Rad Laboratories, Hercules, CA), as described previously [26], [28]–[30], [45], [54]. Primer sets and PCR conditions are summarized in Table 1.

**Table 1:** Human and murine Primer pairs as used for real-time RT-PCR.

Human	Forward (5′-3′)	Reverse (5′-3′)	Product [bp]	Temp [C°]
**ß-actin**	GGTGGCTTTTAGGATGGCAAG	ACTGGAACGGTGAAGGTGACAG	**161**	**58–61**
**TLR2**	GGAGCTGAAGAACTTCAATC	TTGCACCACTCACTCTTC	**159**	**58**
**TLR6**	CAGAGTGAGTGGTGCCATTA	GCCTTCAGCTTGTGGTACTT	**137**	**61**
**TLR10**	AGGTGCAGTGGCTCACTCTT	TTCACCATGTTGGCCAGGAT	**100**	**58**

Murine	Forward (5′-3′)	Reverse (5′-3′)	Product [bp]	Temp [C°]
**ß-actin**	CGAGCGTGGCTACAGCTTCA	AGGAAGAGGATGCGGCAGTG	**122**	**58–60**
**TLR1**	TGATCTTGTGCCACCCAACA	GCAGGGCATCAAAGGCATTA	**66**	**58**
**TLR2**	TTGCTCCTGCGAACTCCTAT	AGCCTGGTGACATTCCAAGA	**157**	**58**
**TLR3**	TAGACTGCATCGCCTGCTAA	AAGCAGCCAGAAGCAGAACT	**122**	**59**
**TLR4**	TCCGGAAGTTCACATAGCTG	TCCATCTCACAAGGCATGTC	**163**	**58**
**TLR5**	AGCTGGTGTTCAAGGACAAG	CCTGGATGTTGGAGATATGG	**210**	**57**
**TLR6**	TCTGGGATAGCCTCTGCAACA	GGCGCAAACAAAGTGGAAAC	**71**	**60**
**TLR7**	TACCAGGACAGCCAGTTCTA	AGGAGCCTCTGATGAGACAA	**136**	**58**
**TLR8**	TGCGCTACCACCTTGAAGAG	CACTGGTTCCAGGAGGATGA	**248**	**58**
**TLR9**	ACCTCAGCCACAACATTCTC	TGCACCTCCAACAGTAAGTC	**142**	**58**
**TLR11**	GGCAGAGGCTCCATAGTTAC	CCGTCTCTTCAGTTGCTCAC	**116**	**58**
**TLR12**	CGCTTATGTCCAGGACAAGA	GAGGAGAGGCAAGCCAATTA	**144**	**58**
**TLR13**	AGCCTAGCTGCCTGGAAGAA	AGCTCAACAGGATGGAGAGT	**100**	**58**

### Immunoblotting experiments

Immunoblotting experiments were performed as described previously[44], [45], [47], [49], [54], [55]. In short, HMEC-1 were grown to confluence on 100-mm dishes and exposed to indicated experimental conditions. The monolayers were lysed for 10 min in 300 µl lysis buffer (150 mM NaCl, 25 mM Tris, pH 8.0, 5 mM EDTA, 2% Triton X-100, and 10% mammalian tissue protease inhibitor cocktail; Sigma-Aldrich), scraped and collected into microfuge tubes. After spinning at 14,000 *g* to remove cell debris, the pellet was discarded. Proteins were solublized in nonreducing Laemmli sample buffer and heated to 70°C for 10 min. Samples were resolved on a 12% polyacrylamide gel and transferred to nitrocellulose membranes. The membranes were blocked for 1 h at room temperature in TBS supplemented with 0.05% Tween 20 and 3% nonfat dry milk. The membranes were incubated either with polyclonal rabbit anti-TLR2 (Acris, 2 µg/ml) or with polyclonal rabbit anti-TLR6 (Abcam, 2 µg/ml) in PBS-T supplemented with 0,05% Tween 20 and 3% nonfat dry milk for 1h at 4°C over night, followed by 10 min washes in PBS-T. The membranes were incubated in goat anti-rabbit (Pierce, 200 ng/ml) and conjugated to horseradish peroxidase for 1 h at room temperature. The wash was repeated and proteins were detected by enhanced chemiluminescence. To control for protein loading, blots were stripped and re-probed for ß-actin using a mouse monoclonal anti-human ß-actin antibody (Sigma-Aldrich).

### TLR stimulation and IL6 ELISA

Similar numbers of HMEC-1 (∼10^5^ cells/well) were seeded in a 24-well plate and grown to confluence (2–3 days). After adding new media, cell monolayers were stimulated with specific TLR ligands (P3C, TLR2 ligand) from EMC microcollections GmbH, Tuebingen, Germany; FSL-1 (synthetic, TLR2/6 ligand) from InVivoGen, San Diego, CA, USA) with indicated ligand concentrations (1, 10 and 100 ng/ml). Cells were exposed to hypoxia and normoxia for 24h. Afterwards, supernatants were harvested, centrifuged to remove cellular debris, and stored at −70°C until assayed by ELISA. Human IL-6 was quantified using matched antibody pairs from R&D Systems in an ELISA according to the manufactures instructions.

### Chromatine immunoprecipitation (ChIP) assay

ChIP assays were performed using HMEC-1 subjected to normoxia or hypoxia [30]. In brief, 2×10^7^ cells were fixed with 1% paraformaldehyde for 10 min. Cross-linking was stopped by the addition of 125 mM glycine, and chromatin derived from isolated nuclei was sheared using a F550 micro-tip cell sonicator (Fisher Scientific). After centrifugation, supernatants containing sheared chromatin were incubated for 4 h with 5 µg of anti–HIF-1α antibody or IgG control (Upstate Biotechnology) as negative control. Protein A sepharose was added and the incubation continued overnight at 4°C. Immune complexes were washed extensively and eluted from the protein A sepharose. The supernatants were transferred to a new tube, and 1 µg/µl of RNase was added and incubated for 5 h at 67°C. Samples were frozen at −80°C and 60 µg/µl proteinase K was added and incubated for 2 h at 45°C. Next, DNA was purified and extracted using Nucleo Spin Extract II kit (Macherey& Nagel, Dueren, Germany) and analyzed by PCR. 1 µl of sample was used for each PCR reaction. The sequences of the TLR2 and TLR6 promoter-specific primers spanning the putative HIF-1–binding regions were as follows. TLR2: sense, 5′-TCAGCGCGAGGTCCAGAGTT -3′ and antisense, 5′-TCCGAGCAGTCACCTGAGAG -3′. The size of the amplified product resulting from the use of this primer pair was 320 bp. TLR6: sense, 5′- AAGATGAGCCAGAGGTGAAG-3′ and antisense, 5′- GCAAGCAGCAGACACATCAA-3′. The size of the amplified product resulting from the use of this primer pair was 285 bp. The primer sets were amplified using increasing numbers of cycles of 94°C for 1 min, 56°C for 2 min, 72°C for 4 min, and a final extension of 72°C for 7 min. The PCR transcripts were visualized on a 2% agarose gel containing 5 µg/ml of ethidium bromide.

### In vivo hypoxia model and immunohistochemistry

Colonic mucosal scraping (enriched in epithelial cells) were obtained from 6–8 wk-old conditional HIF-1α mutant mice or littermate controls, as described previously [31]. Organs were obtained from C57BL/6 mice exposed either to normobaric hypoxia (8% O_2_ and 92% N_2_) or room temperature air for 6 h (n = 6 animals per condition). After hypoxia/normoxia exposure, the animals were killed and organs were harvested. For RT-PCR analysis scrapings and organs tissue were homogenized in RNAlater (QUIAGEN) using a 22-gauge syringe (Becton-Dickinson) and Quiashredder column (QUIAGEN). Further analysis of TLR2 and TLR6 mRNA fold changes were performed as described above. For immunohistochemistry organs were harvested and collected in a tube containing Tissue Tec freezing media and were then subsequently shock frozen. Frozen tissue were cut to 5-µm sections and placed on a glas slide. After fixation with acetone for 10 min and washing in PBS for 10 min sections were blocked in TBST supplemented with 0.05% Tween 20 and 5% of human serum at room temperature for 30 min. After blocking sections were incubated either with monoclonal mouse anti-TLR2 (eBioscience, 1∶50–1∶200 dilutions), or with polyclonal goat anti TLR6 (Santa Cruz Biotechnology Inc., 1∶50–1∶200 dilutions) in PBS-T supplemented with 0,05% Tween 20 and 5% of human serum for 1 h at room temperature, followed by washing twice in PBS. Alexa Fluor 488-conjugated goat anti-mouse (TLR2 and IgG) or donkey anti-goat (TLR6) were used as secondary antibodies (Invitrogen, 1∶5000 for 60 min at room temperature). After washing twice with PBS samples were mounted (ProLong Gold antifade reagent, Invitrogen) and assessed within the next 24 h using a laser-scanning confocal microscope (Leica). Single-plane optical slices were then processed for double-labelled cells using identical laser and standardized microscope settings and exported to Adobe Photoshop 5.0LE (TIFF). Representative results are shown for the experiment for each condition. These protocols were in accordance with National Institutes of Health guidelines for use of live animals and were approved by the Institutional Animal Care and Use Committee at University of Colorado Health Science Center in Denver.

### Data analysis

Data were compared by 2-factor ANOVA or Student's *t* test, where appropriate. Values are expressed as the mean±SEM from at least three separate experiments per condition. P<0.05 was considered statistically significant.
